# Particle-Mediated Histotripsy for the Targeted Treatment of Intraluminal Biofilms in Catheter-Based Medical Devices

**DOI:** 10.34133/2022/9826279

**Published:** 2022-07-05

**Authors:** Christopher Childers, Connor Edsall, Isabelle Mehochko, Waleed Mustafa, Yasemin Yuksel Durmaz, Alexander L. Klibanov, Jayasimha Rao, Eli Vlaisavljevich

**Affiliations:** ^1^Virginia Tech Carilion School of Medicine, USA; ^2^Department of Biomedical Engineering and Mechanics, Virginia Tech, USA; ^3^Department of Biomedical Engineering, Istanbul Medipol University, Turkey; ^4^Division of Cardiovascular Medicine (Department of Medicine) and Robert M. Berne Cardiovascular Research Center at University of Virginia School of Medicine, University of Virginia, USA; ^5^Department of Medicine, Division of Infectious Diseases, Virginia Tech Carilion School of Medicine, USA; ^6^ICTAS Center for Engineered Health, Virginia Polytechnic Institute and State University, USA

## Abstract

*Objective*. This paper is an initial work towards developing particle-mediated histotripsy (PMH) as a novel method of treating catheter-based medical device (CBMD) intraluminal biofilms. *Impact Statement*. CBMDs commonly become infected with bacterial biofilms leading to medical device failure, infection, and adverse patient outcomes. *Introduction*. Histotripsy is a noninvasive focused ultrasound ablation method that was recently proposed as a novel method to remove intraluminal biofilms. Here, we explore the potential of combining histotripsy with acoustically active particles to develop a PMH approach that can noninvasively remove biofilms without the need for high acoustic pressures or real-time image guidance for targeting. *Methods*. Histotripsy cavitation thresholds in catheters containing either gas-filled microbubbles (MBs) or fluid-filled nanocones (NCs) were determined. The ability of these particles to sustain cavitation over multiple ultrasound pulses was tested after a series of histotripsy exposures. Next, the ability of PMH to generate selective intraluminal cavitation without generating extraluminal cavitation was tested. Finally, the biofilm ablation and bactericidal capabilities of PMH were tested using both MBs and NCs. *Results*. PMH significantly reduced the histotripsy cavitation threshold, allowing for selective luminal cavitation for both MBs and NCs. Results further showed PMH successfully removed intraluminal biofilms in Tygon catheters. Finally, results from bactericidal experiments showed minimal reduction in bacteria viability. *Conclusion*. The results of this study demonstrate the potential for PMH to provide a new modality for removing bacterial biofilms from CBMDs and suggest that additional work is warranted to develop histotripsy and PMH for treatment of CBMD intraluminal biofilms.

## 1. Background

Catheter-based medical devices (CBMDs) are thin tubes commonly made of medical-grade materials that serve a variety of functions including urine removal, medication delivery, and dialysis [1–3]. Each year, more than 30 million urinary catheters, 5 million central venous catheters, and 450,000 vascular grafts are inserted in the United States alone [4]. These CBMDs commonly become infected with bacterial biofilms [5–7]. The pathogenesis of biofilm formation has been previously described by planktonic bacteria binding to the abiotic catheter wall, and once adhered, the bacteria secrete exopolysaccharides forming a biofilm. Once mature, biofilms are difficult to eradicate and can allow bacteria to break off and seed additional sites including the bloodstream [6, 8, 9]. The feared long-term sequelae of biofilm formation is catheter-related bloodstream infections (CRBSI) which are defined by bacteremia associated with a CBMD [10]. CBMD biofilm formation and subsequent infection have been shown to lengthen hospital stays, increase patient and hospital costs, and raise patient mortality and morbidity [8, 9, 11–14]. Thus, there is a significant need for the development of a method for selectively targeting and eradicating established luminal biofilms for preventing or treating CBMD biofilms.

Recently, our team conducted a study investigating histotripsy for the treatment of luminal biofilms [15]. Histotripsy is an image-guided and nonthermal focused ultrasound ablation method that uses high-pressure acoustic pulses to noninvasively ablate biofilms and tissues through acoustic cavitation [16, 17]. Previously, histotripsy has been shown to ablate eukaryotic tissues by creating cavitation “bubble clouds” which destroyed the tissues leaving only an acellular homogenate behind [16, 18, 19]. The ablative properties of histotripsy have been utilized in preclinical and clinical settings, most notably for liver tumor ablation [18–21]. In addition to eukaryotic tissues such as tumors, studies have shown that histotripsy will disrupt bacterial biofilms on surgical meshes, microscope slides, and graphic discs [22–26]. More recently, our lab showed that conventional histotripsy methods could be used to precisely target the lumen of urinary catheters, remove luminal biofilms, and kill suspended bacteria [15].

This work builds upon our previous study that established the feasibility of utilizing conventional histotripsy methods for the treatment of luminal biofilms [15]. Although the results of that study were promising, conventional histotripsy requires high peak negative pressures (p−) often greater than 25 MPa to create cavitation bubble clouds. In addition, conventional histotripsy requires real-time image guidance to achieve precise targeting. In the clinical setting, this approach would require a skilled clinician to perform the procedures, such as an interventional radiologist or other clinician with training in image-guided interventions. To overcome these potential limitations, this study investigates particle-mediated histotripsy (PMH) methods that utilize externally injected particles, such as fluid-filled nanoparticles or gas-filled microbubbles (MBs), to reduce the histotripsy cavitation threshold following an approach used previously for oncology applications [27–30]. Prior work has shown that perfluoropentane (PFP) or perfluorohexane (PFH) nanodroplets can significantly reduce the histotripsy cavitation threshold, with reported p− thresholds of ~9-11 MPa at 500 kHz [29, 31, 32]. Similarly, our team has recently developed PFH “nanocones” (NCs) as an improved histotripsy agent with similar functionality to the PFH nanodroplets but with a smaller size (~40 nm vs. ~200 nm), high stability, and a simple synthesis process that merely involves the mixing of two FDA-approved compounds [27, 28]. Finally, in addition to these fluid-filled nanoparticles, prior work has shown that gas-filled MB can be used to directly seed cavitation, with recent work in our lab showing the p− threshold for generating histotripsy bubble clouds from MBs to be ~4-5 MPa [33]. These MBs, which are commonly used as FDA-approved contrast agents for ultrasound imaging [34], consist of small pockets of gas typically surrounded by phospholipid molecules, polymers, or other shells. Using these fluid-filled or gas-filled particles, histotripsy cavitation can be selectively generated only in regions containing the nucleating agent, allowing for a targeted ablation method that can be applied without the need for extremely powerful histotripsy transducers and image-guided targeting approaches [29].

In this study, we investigate the feasibility of using PMH for the treatment of luminal biofilms in a Tygon catheter mimic model. Unlike conventional high-pressure histotripsy methods, PMH would allow for a more convenient tool to noninvasively prevent or remove catheter-associated biofilms without the need for real-time image guidance and large transducers capable of generating high acoustic pressures. If viable, PMH could be utilized to treat completely formed biofilms or could be used prophylactically on newly forming biofilms by removing and killing bacteria at earlier time points before biofilm maturation. Here, we use PMH to treat completely formed biofilms to simulate the worst-case scenario of these two choices. To test our hypothesis that PMH can be utilized for the ablation of luminal biofilms in CBMDs, we conducted a series of experiments to compare the cavitation thresholds inside catheters containing no particles (conventional histotripsy) as well as NCs or MBs at a range of concentrations. In addition, we tested the feasibility of selectively removing biofilms and killing suspended bacteria with PMH using both NCs and MBs.

## 2. Results

### 2.1. Cavitation Threshold of Particle-Mediated Histotripsy in Tygon Catheters

**Figure 1:**
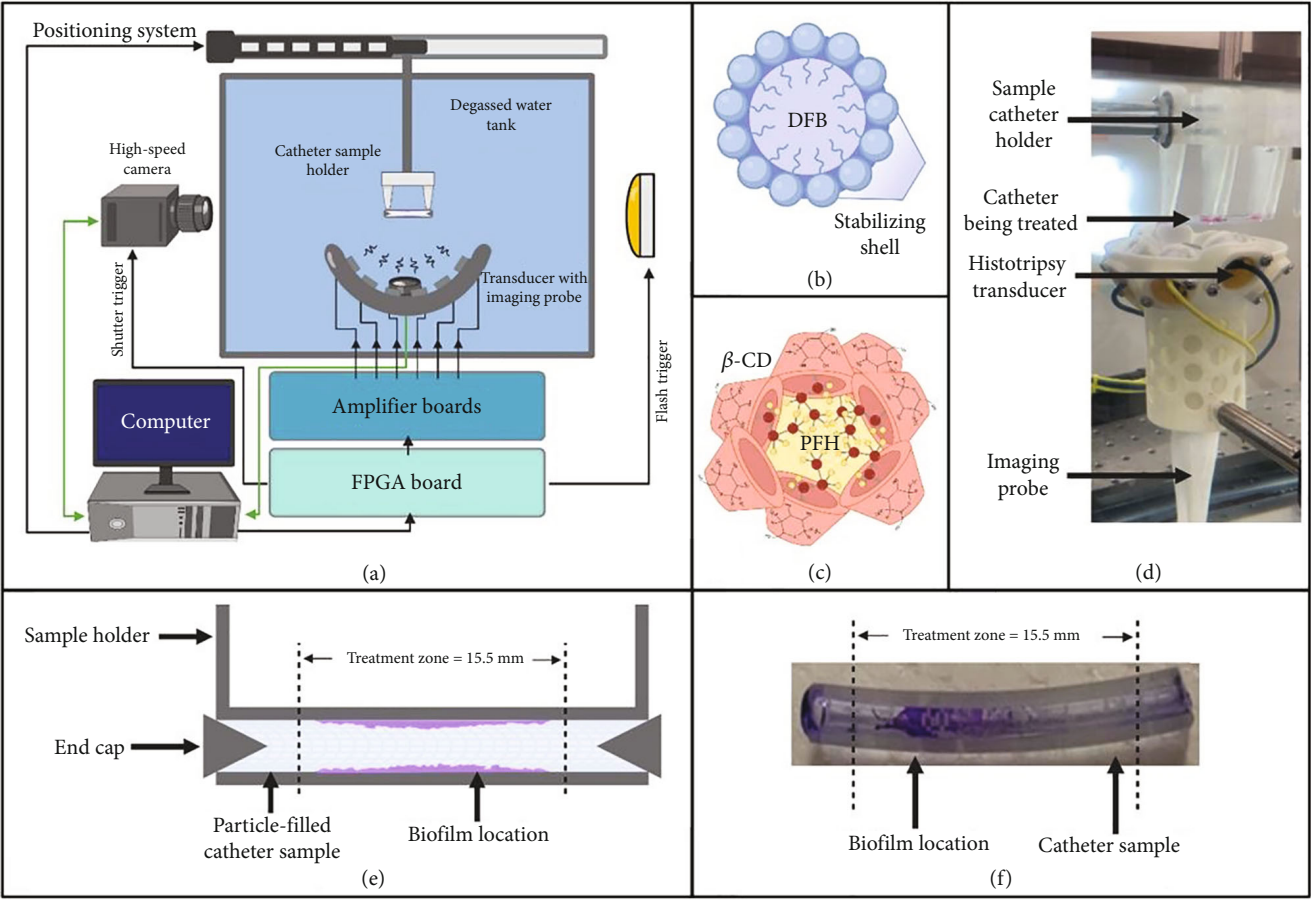
Experimental set-up. (a) Schematic showing 3D positioning system used for precise movement of samples through the focus of a stationary histotripsy transducer with coaxial ultrasound imaging and perpendicular high-speed optical imaging. (b) Molecular structure of a microbubble. (c) Molecular structure of a nanocone cluster. (d) Visual image of experimental set-up. (e) Graphical depiction of catheter with biofilm and 15.5 mm treatment zone. (f) Visual image of catheter with crystal violet-stained biofilm.

**Figure 2:**
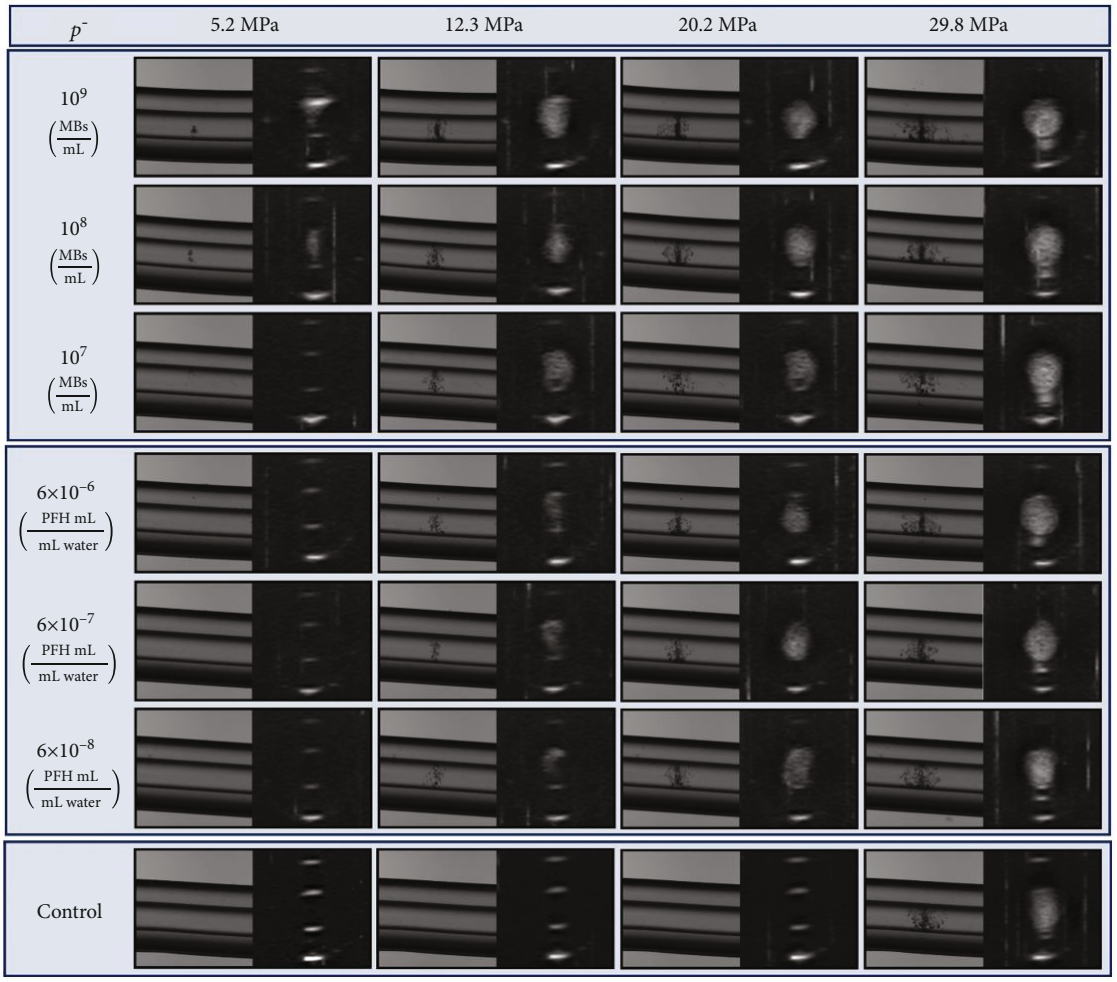
MB-mediated cavitation. Coaxial ultrasound imaging and perpendicular optical imaging of cavitation bubble clouds generated inside catheters containing MBs or NCs at a range of concentrations and at select pressures.

In this study, we investigated a novel PMH approach for the treatment of luminal biofilms inside CBMDs. In the first part of this work, we hypothesized that PMH could be utilized to reduce cavitation pressures to enable selective cavitation inside of a catheter lumen. To test this hypothesis, the pressure required to generate histotripsy cavitation clouds within Tygon catheters was investigated for samples containing MBs, NCs, and no particles (conventional histotripsy control) (Figure 1). Results showed significantly reduced cavitation thresholds for PMH compared to control samples, with robust cavitation clouds visualized as dynamically changing hyperechoic regions within the catheter lumen on ultrasound imaging and dark, well-confined bubble clouds within the catheter lumen on optical imaging. Bubble clouds observed inside the catheter lumens for both MBs and NCs (Figure 2) were visualized at pressures significantly below the histotripsy intrinsic threshold, matching our hypothesis. At the pressure levels directly above the cavitation threshold for each particle type, the cavitation bubble clouds were observed as small clouds formed inside the center of the catheter lumen. As the pressure was increased above the respective cavitation thresholds, the size of the bubble clouds increased to fill the entire catheter lumen in both MB and NC samples (Figure 2).

**Figure 3:**
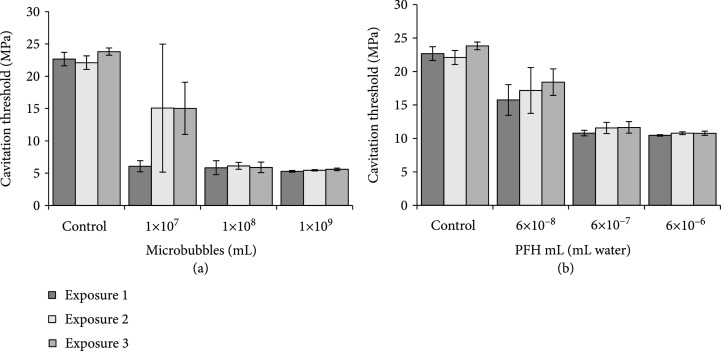
MB and NC cavitation threshold. Plot shows the pressure require to generate PMH cavitation within the lumen of the Tygon catheter mimic. Cavitation threshold denotes initial creation of cavitation for (a) MB and (b) NC.

Comparing the cavitation threshold for samples containing MBs at a range of concentrations showed a cavitation threshold of 6.1±0.4 MPa for the first exposure at the lowest concentration tested, $1\times 10^{7}$ MBs/mL (Figure 3(a)). This was significantly lower than the exposure paired control sample, which showed a cavitation threshold of 22.7±0.5 MPa. Tukey post hoc analysis showed statistical significance between control and $1\times 10^{7}$ MBs/mL samples (p<0.05). When the cavitation threshold was measured for exposures 2 and 3 in the $1\times 10^{7}$ MBs per mL samples, the threshold increased to 15.1±4.5, and 15.0±1.8 MPa, with the respective controls at 21.1±0.5 MPa, and 23.8±0.3 MPa (Figure 3(a)). Again, Tukey post hoc analysis showed statistical significance between control and $1\times 10^{7}$ MBs/mL samples within exposures two and three (p<0.05). This finding matches previous work showing that MBs, which are used to directly seed cavitation, are destroyed by the histotripsy process and do not serve as repeatable cavitation nuclei over the course of multiple pulses [28]. When the MB concentration was increased to $1\times 10^{8}$ MBs/mL, the cavitation results were consistent with a cavitation threshold of 5.8±0.5 MPa, 6.1±0.2 MPa, and 5.9±0.4 MPa for exposures 1, 2, and 3, respectively, each showing statistical significance to the respective exposure control on Tukey post hoc analysis (p<0.05). Similar results were observed at the highest concentration of MBs, $1\times 10^{9}$ MBs/mL, which showed the cavitation threshold to be 5.3±0.1 MPa, 5.5±0.0 MPa, and 5.6±0.1 MPa for the three exposures, again each showing statistical significance to the respective exposure control on Tukey post hoc analysis (p<0.05) (Figure 3(a)). Further statistical analysis using Tukey post hoc testing of the two-way ANOVA showed no statistical intrasample difference across exposures 1, 2, and 3 within the control, $1\times 10^{8}$ MBs/mL and $1\times 10^{9}$ MBs/mL samples (p>0.05). However, within the $1\times 10^{7}$ MBs/mL samples, statistical significance was found between exposures 1 and 2 along with 1 and 3 (p<0.05). Exposures 2 and 3 remained not statistically significant (p>0.05). Together, these results suggest that a sufficient amount of MBs were remaining after the initial exposures to maintain a reduced cavitation threshold at the higher concentrations of $1\times 10^{8}$ and $1\times 10^{9}$ MBs per mL despite the potentially deleterious effects of the previous pulses.

Comparing the cavitation threshold for samples containing NCs showed a concentration-dependent effect for reducing the histotripsy cavitation threshold, matching a previous study [27]. For samples containing NCs at the lowest concentration of $6\times 10^{-8}$ mL PFH/mL water, the cavitation threshold was measured to be 15.7±2.3 MPa, 17.2±3.4 MPa, and 18.4±2.0 MPa for exposures 1, 2, and 3, respectively (Figure 3(b)). Although the reduction in the cavitation threshold was smaller than what was observed for MBs (as well as the higher concentration NC samples), the decrease in the cavitation threshold was still significant in comparison to the respective exposure control samples on Tukey post hoc analysis (p<0.05) (Figure 3(b)). These findings are consistent with previous studies showing that NCs can be utilized as sustainable cavitation nuclei over the course of multiple pulses [33] and support our hypothesis that the NCs may offer a more consistent cavitation reduction profile compared to the MBs due to their stable molecular structure and their mechanism of reducing the cavitation threshold (PFH fluid vs. trapped gas nuclei). As the concentration of NCs was increased to $6\times 10^{-7}$ mL PFH/mL water, the cavitation threshold was measured to be 10.8±0.4 MPa, 11.6±0.8 MPa, and 11.7±0.9 MPa for the three exposures and maintained statistical significance to each respective exposure control on Tukey post hoc analysis (p<0.05) (Figure 3(b)). Similarly, the cavitation threshold for samples with NCs at $6\times 10^{-6}$ mL PFH/mL water was found to be 10.5±0.1 MPa, 10.8±0.2 MPa, and 10.8±0.3 MPa again, statistical significance was maintained to each respective exposure control on Tukey post hoc analysis (p<0.05) (Figure 3(b)). Tukey post hoc testing of the two-way ANOVA showed no statistical intrasample difference across exposures 1, 2, and 3 within the control and each concentration tested (p>0.05).

### 2.2. Selective Luminal Cavitation Using Particle-Mediated Histotripsy

**Figure 4:**
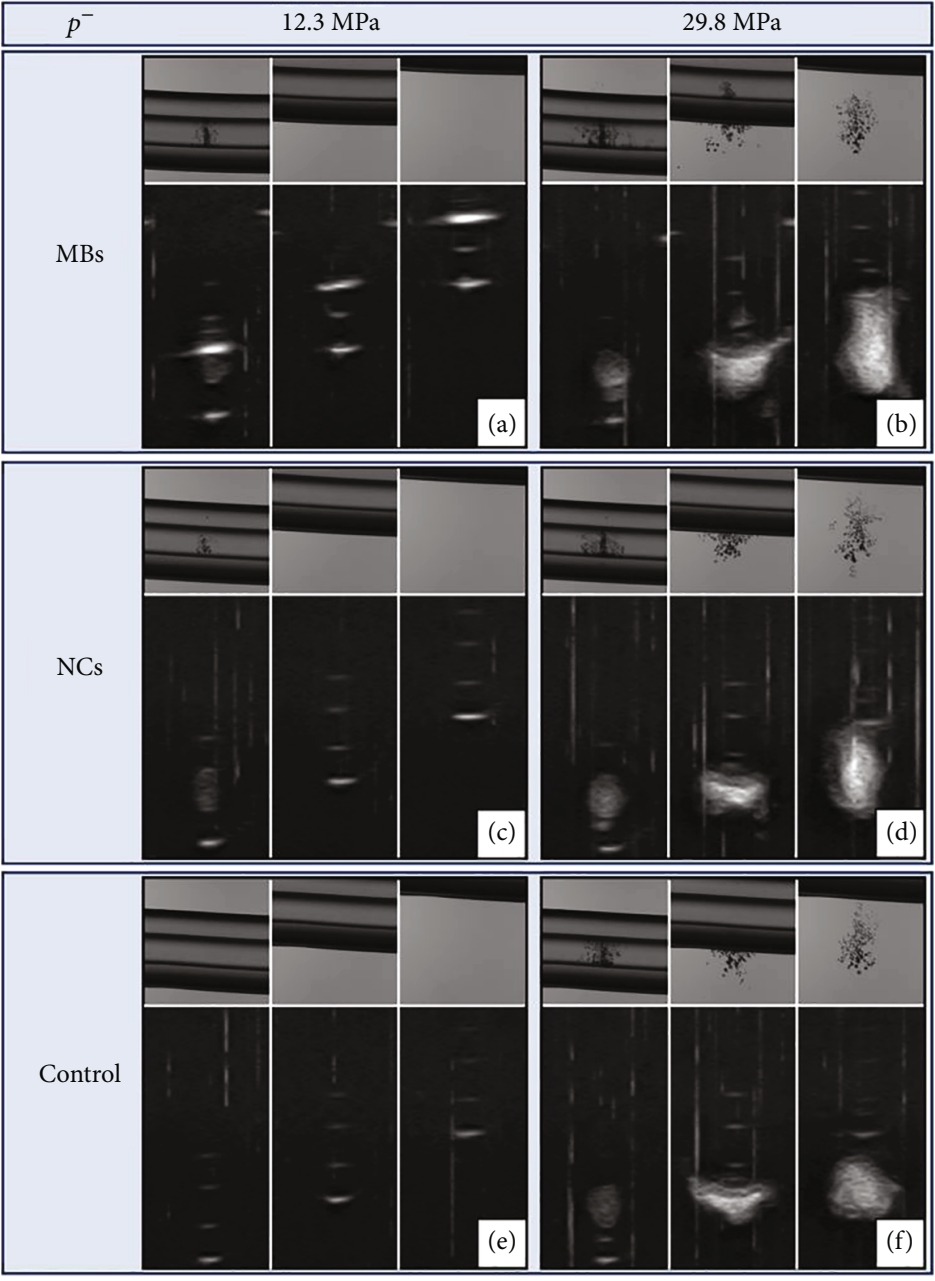
MB-mediated selective cavitation. Images showing various MB (10^9^ Bubble/mL) and NC ($6\times 10^{-6}$ PFH mL/mL water) concentrations at various pressures with luminal cavitation only the focus covers the lumen denoting selective cavitation.

To test the hypothesis that PMH can create selective luminal cavitation within catheters without generating any cavitation outside of the catheters, a second set of cavitation experiments was conducted. Results showed well-defined bubble clouds formed inside the catheters for both MB and NC filled samples exposed to ultrasound at a p− of 12.3 MPa on both ultrasound and optical imaging when the transducer focus was aligned to the center of the catheter lumen (Figures 4(a) and 4(c)). In contrast, control samples showed no detectable cavitation clouds at 12.3 MPa even when the focus was aligned directly to the center of the catheter (Figure 4(e)). When the catheter samples were moved 3 mm or 6 mm vertically from the center of the transducer focus, cavitation was no longer observed inside the catheter lumen nor extraluminally at the focal location for any samples at 12.3 MPa (Figures 4(a) and 4(c)). These data demonstrate the selectivity of PMH methods for generating intraluminal cavitation. For comparison, a final set of control experiments was conducted at a p− of 29.8 MPa in the same focal positions. These experiments showed cavitation was generated at the focus inside the catheter when the catheter was aligned with the focus as well as when the catheter samples were positioned 3 mm or 6 mm vertically from the focus center, in which case the cavitation cloud was generated outside of the catheter (Figures 4(b), 4(d), and 4(f)). This finding is expected for the pressure levels used in conventional histotripsy methods that are not selective to the fluid inside of the catheter.

### 2.3. Particle-Mediated Histotripsy Biofilm Removal

**Figure 5:**
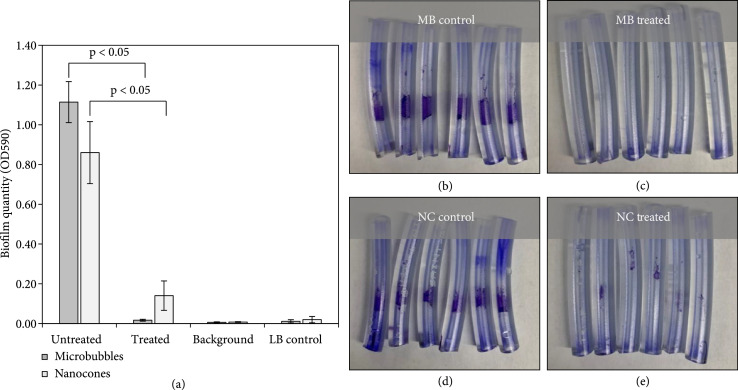
MB Biofilm Removal. MB ($1\times 10^{9}$ microbubbles/mL), and NC ($6\times 10^{-6}$ PFH mL/mL water) biofilm removal at 12.3 MPa 200 PRF with focus increments one 0.1 mm for a total treatment zone of 15.5 mm with 500 pulses per increment. (a) Optical density at 590 nm of solubilize CV stain. (b) Untreated. (c) MB treated. (d) Untreated. (e) NC treated.

Based on the cavitation experiment results, we hypothesized that PMH could be used to remove luminal biofilms with a similar efficacy to what was shown in our prior study with conventional histotripsy, but at a reduced pressure. Results from the biofilm experiments showed that MB-mediated histotripsy (MMH) applied at a p− of 12.3 MPa generated a cavitation cloud that completely filled the catheter lumen throughout the duration of the treatment, which consisted of a single scan across the catheter. The luminal bubble cloud was observed in real-time during the treatment using both optical and ultrasound imaging, matching the results observed in the cavitation experiments previously described. Quantifying biofilm removal after treatment showed that MMH resulted in a significant reduction in the visible crystal violet staining as well as quantified biofilm using spectrophotometry (Figure 5). More specifically, the mean untreated catheter OD590 was 1.11±0.1, whereas the mean MMH treated catheter was 0.02±0.00. For comparison, the mean background catheter and LB control OD590 for these experiments were 0.01±0.00 and 0.01±0.01, respectively. These data indicated that MMH treatment significantly (p<0.05) reduced the mean OD590 by 1.10, or 98% after only a single scan through the catheter (Figure 5(a)). Upon visual inspection of the untreated catheters, crystal violet-stained biofilm was clearly seen within the catheter lumen (Figure 5(b)). However, after treatment with MMH, there was a visible lack of crystal violet staining within the lumen of the catheters, indicating biofilm removal (Figure 5(c)). Further, control samples showed that the catheters did not contribute to crystal violet staining as seen visually by the lack of staining and near-zero background OD signal (Figure 5(a)). Finally, LB control catheters did not contribute to staining, indicating LB media sterility (Figure 5(a)).

Biofilm removal experiments using NC-mediated histotripsy (NMH) applied at a p− of 12.3 MPa resulted in cavitation clouds generated inside the treated catheters during treatment, as expected. However, the bubble clouds did not fill the entire cross-sectional lumen throughout the treatment. Instead, ultrasound imaging showed the bubble cloud covered only a portion of the cross-sectional circumference of the lumen at 12.3 MPa as seen in Figure 2 at 12.3 MPa with $6\times 10^{-6}$ mL PFH/mL water NCs. This finding, which is consistent with the higher cavitation threshold for the NCs compared to the MBs, suggests that NMH may have reduced efficacy compared to MMH when applied at the same pressure level due to a smaller bubble cloud being generated in the lumen. Although the pressure was kept at 12.3 MPa for the experiments in this study to maintain consistency across the PMH experiments with both particle types, higher pressures could be utilized in future studies using NCs to optimize bubble cloud characteristics for more effective biofilm removal. After treatment with NMH at 12.3 MPa, results showed the treated catheter OD590 was 0.14±0.07, which was significantly (p<0.05) lower than the untreated catheter OD590 of 0.86±0.16. The mean background catheter and LB control OD590 for these experiments were 0.01±0.00 and 0.02±0.02, respectively. Similar to the results from MB experiments, visual inspection of the untreated catheters showed a crystal violet-stained biofilm within the catheter lumen (Figure 5(d)). For catheters treated with NMH, there was a visible reduction in crystal violet staining within the lumen (Figure 5(e)). However, small patches of remaining crystal about stained biofilm remained, indicating only partial biofilm removal was achieved after the single scan treatment (Figure 5(e)). These results were consistent with the observation that the cavitation created by NMH did not completely fill the catheter lumen, as discussed above. Control samples again showed that the catheters did not contribute to crystal violet staining, as seen visually by the lack of staining and near-zero background OD signal (Figure 5(a)). Finally, LB control catheters did not contribute to staining indicating LB media sterility (Figure 5(a)).

### 2.4. Particle-Mediated Histotripsy Bactericidal Effects

A final set of experiments was conducted to test the ability of PMH to kill suspended bacteria. Clinically, a desirable PMH therapy would allow for both the removal of bacterial biofilms as well as the destruction of planktonic bacteria released after biofilm removal. During these experiments, the bactericidal effect of PMH was compared for samples treated with 1, 2, 4, and 6 scans through the catheter. Cavitation was observed within the lumen of the catheter during the first two scans for samples containing both MBs and NCs. However, cavitation activity significantly decreased within the lumen of the catheters for subsequent scans. For instance, minimal posterior wall cavitation was noted for the MB group during scan numbers 4 and 6, and no cavitation activity was noted in the NC group during scan numbers 4 and 6 (see Supplemental (available here)).

**Figure 6:**
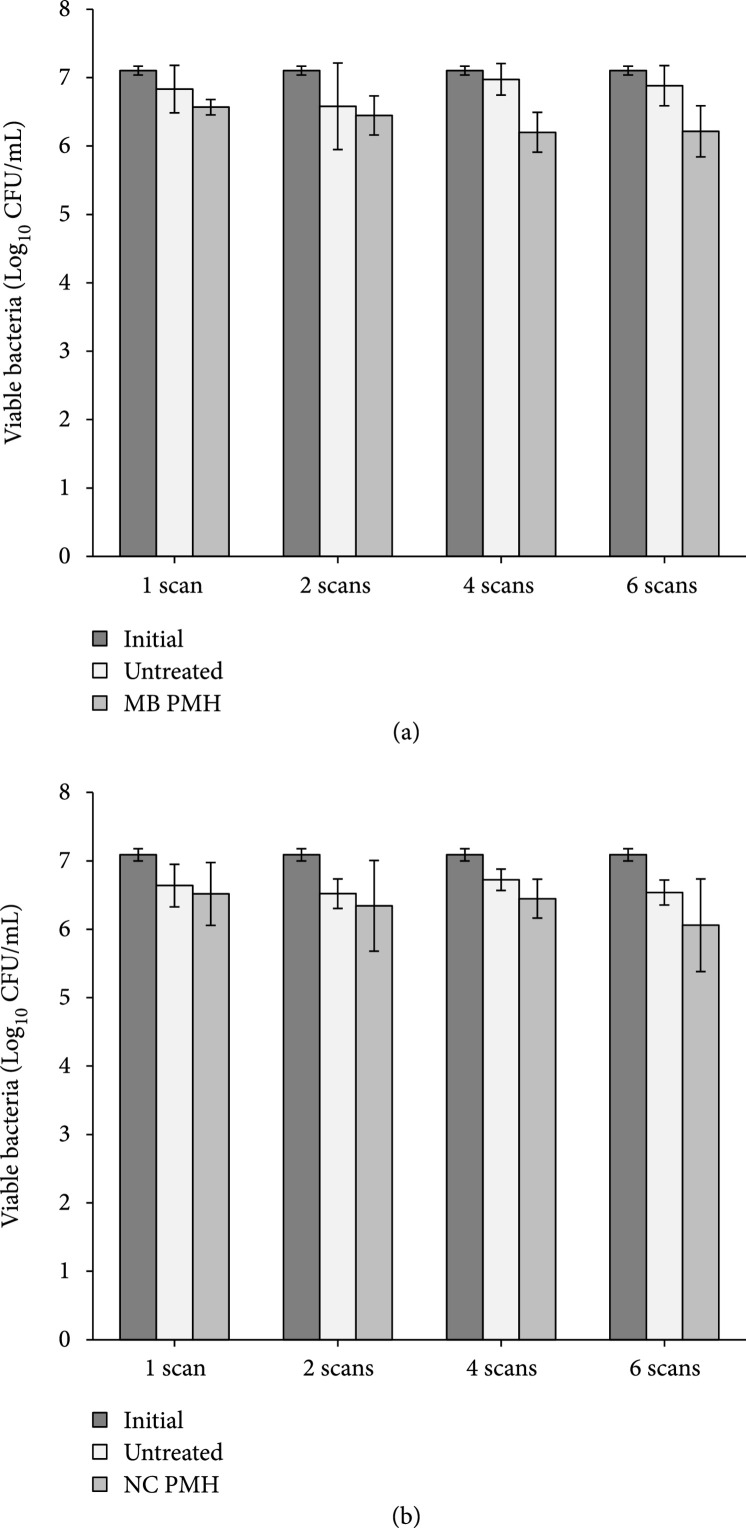
MB and NC bactericidal effects on suspended bacteria. Plot shows CFU/mL after treatment, and control Tygon catheter mimics filled with suspended PA14 and either $1\times 10^{9}$ microbubbles/mL (a) or $6\times 10^{-6}$ PFH mL/mL water (b). Scans indicate the number of passes the treatment transducer passed through the catheter mimic.

Analysis of CFU/mL after PMH showed only a small bactericidal effect, with a significant number of viable bacteria remaining after increasing scans across the Tygon catheter lumen (Figure 6). More specifically, results showed a 0.26±0.26 and 0.12±0.35 log10 reduction in viable bacteria after a single scan of the catheter with MB and NMH, respectively. Results showed a minor increase in the bactericidal effect of PMH for multiple scan treatments, using both MBs and NCs. The results of increasing scan number for MMH were 0.13±0.47, 0.77±0.27, and 0.67±0.21 log10 reduction in viable bacteria after 2, 4, and 6 scans across the catheter, respectively (Figure 6(a)). The results of increasing scan number for NMH were 0.18±0.66, 0.28±0.29, and 0.48±0.72 log10 reduction in viable bacteria after 2, 4, and 6 scans across the catheter, respectively (Figure 6(b)). Comparisons of these treatments showed a statistically significant difference between untreated samples and MMH treated catheters for 4 and 6 scan treatments (p<0.05) but no significant different for any of the NMH treated catheters (p>0.05).

## 3. Discussion

### 3.1. Cavitation Threshold of Particle-Mediate Histotripsy in Tygon Catheters

Overall, the implications of the cavitation threshold results are important to the clinical translation of histotripsy-based methods for the treatment of biofilms in CBMDs, particularly catheters located in regions that are difficult to target with conventional histotripsy methods. Results from these experiments support our hypotheses that PMH can consistently generate well-confined bubble clouds in catheters at lower pressures than conventional histotripsy if a high enough particle concentration is used. Further, these results correlate with previous data that suggests PMH cavitation cloud size is a function of both applied pressure and particle concentration [27]. Although our prior work has shown that conventional histotripsy can be successful in the treatment of catheter-associated biofilms, the direct implantation of histotripsy for this application would likely face technical and practical challenges before clinical translation. For instance, histotripsy often requires relatively large, focused ultrasound transducers to generate in situ peak negative pressures >25-30 MPa, real-time ultrasound imaging for precise targeting, and technical expertise by a trained user (i.e., interventional radiologist) to safely apply the therapy. The PMH approach established by these experiments represents an alternative method for applying histotripsy without the above limitations, representing a significant advantage for delivering histotripsy reliably to the inside of catheters without the need for large devices and extensive technical expertise. Due to the lower cavitation threshold, it is expected that PMH could be applied with a small, easy-to-use handheld ultrasound probe by health care professionals in a multitude of settings. A few limitations with this study exist. One limitation was that high-speed imaging of the bubble clouds only visualized a single image of each pulse and did not measure the spectral characteristics of the cavitation. Future work should therefore investigate the bubble cloud dynamics in more detail to more fully characterize the cavitation dynamics in this therapy. Additionally, although statistical significance was achieved on Tukey post hoc analysis, threshold testing of the $1\times 10^{7}$ MBs/mL samples showed significant threshold variability with increasing exposures which warranted further investigation (see Supplemental).

### 3.2. Selective Luminal Cavitation Using Particle-Mediated Histotripsy

The results of the selective luminal cavitation experiments provide direct support of our hypothesis that histotripsy can be selectively generated inside catheters when using artificially introduced cavitation nuclei (fluid-filled NCs and gas-filled MBs) without generating any cavitation in the surrounding media (Figure 4). Our previous work demonstrated catheter material, and geometry have a direct effect on the intraluminal cavitation threshold; however, here, we controlled for this effect by using control catheters of identical material and geometry [15]. Future experiments are planned to investigate the potential for any synergistic effect of catheter material and geometry with PMH. Regardless, by utilizing PMH as opposed to conventional methods, histotripsy can be reliably applied to the inside of the catheters likely resulting in a higher safety profile and without the need for large histotripsy transducers guided by real-time imaging. Although the safety profile of PMH was not directly tested in these experiments, future *in vivo* animal model studies are planned to evaluate the preclinical safety of this therapy. Overall, this particle-mediated approach allows for a selective application of histotripsy that does not require the same need for precise image guidance. Using this method, a transducer with a large focal region could be rapidly scanned over a large volume containing a CBMD, with cavitation selectively generated inside the catheter lumen. This approach is also expected to enable treatment methods that could be used as repeated therapy options for both the prevention and removal of luminal biofilms in a wide range of patients at risk of acquiring a CBMD infection. Prevention would be accomplished in a prophylactic manner by treating CBMDs that commonly form biofilms to remove any initially adhered bacteria and prevent maturation of a biofilm. Further, these results also support the possibility that PMH could be utilized in outpatient or low-resource settings, as it would require less training and technical expertise in ultrasound-guided interventions to administer.

### 3.3. Particle-Mediated Histotripsy Biofilm Removal

Results from the biofilm removal experiments demonstrate the feasibility of using PMH to generate histotripsy bubble clouds and efficiently remove a bacteria biofilm at pressure levels significantly below the histotripsy intrinsic threshold. When compared to higher pressure conventional histotripsy treatments from our prior work, PMH showed biofilm ablation results nearly equivalent to that of conventional histotripsy [15]. These results demonstrate the feasibility of using PMH for the removal of biofilms associated with CBMDs and demonstrate the advantage of PMH compared to conventional histotripsy by allowing the therapy to be applied selectively inside catheters at lower acoustic pressures. Due to these features, we expect PMH could be used for the safe, precise, and noninvasive removal of luminal biofilms. Although these initial studies demonstrate the feasibility of selective PMH, further studies will need to be conducted to elucidate the clinical safety of this technology. Additionally, further device development will be needed to construct therapy transducers with deeper focal distances to treat deep indwelling CBMDs. The further development of this technology would enable prolonged catheterization without the current risks for CBMDs. Further, as discussed above, PMH could be used without the need for large expensive equipment or specialty training. The reduction of CBMD biofilms from this technology could be used by medical personnel with less training in the outpatient setting and therefore improve patient outcomes and reduce healthcare costs, particularly for CBMDs such as dialysis access lines, central lines, and ventriculoperitoneal shunts that are difficult to treat without removal and replacing which is costly and could cause the patient more harm.

### 3.4. Particle-Mediated Histotripsy Bactericidal Effects

Results from the bactericidal experiments did not support our hypothesis that PMH can kill suspended PA14 in a dose-dependent manner as was previously observed in our studies using conventional histotripsy methods [15]. Although a small reduction in bacteria was seen with PMH, it should be noted that a clinically significant reduction in viable bacteria is on the order of 2 to 3 magnitudes, which was not achieved using the treatment parameters in this study and should be investigated more in future studies. It is possible that the lower pressures used in PMH may have reduced the bactericidal effects of the treatment compared to conventional histotripsy at higher pressures, which would be consistent with prior studies showing a decrease in histotripsy ablation efficiency for PMH methods tested in red blood cell tissue phantoms, likely due that to reduced bubble expansion at the lower pressure levels [33]. When compared with data from our previous work with conventional histotripsy, PMH at a p− of 12.3 MPa showed less bactericidal activity after 1 scan than what was observed for conventional histotripsy at a higher pressure of 47.6 MPa [15]. This suggests that the pressure at which the histotripsy bubble cloud is generated likely plays a role in the extent of the bactericidal effects. However, the observation that cavitation became inconsistent after more than 1 or 2 scans in the PMH studies makes it difficult to conclusively compare the bactericidal effect of PMH to conventional histotripsy methods, since PMH therapy was not able to be delivered throughout the planned treatment. Since histotripsy requires a consistent bubble cloud to be effective, the loss of cavitation with increasing numbers of treatment scans may also explain the minimal bactericidal activity observed here. Additionally, the difference in ablation efficiency between MMH and NMH is likely due at least in part to the difference in treatment pressure above the respective cavitation threshold for each ablation method. In these experiments, we used 12.3 MPa for both ablation methods which corresponds to ~7 (128%) and ~1.8 (16%) MPa above the cavitation threshold for MMH and NMH, respectively. Therefore, it is expected that NMH will generate a smaller bubble cloud and result in a reduced ablation efficacy in comparison to the MMH group, as observed. Additional studies characterizing the change in the cavitation threshold as a function of scan number (Supplemental Data) showed that the cavitation threshold began to rise back near that of intrinsic threshold cavitation as treatments progressed from 1 to 6 scans. Further work will be needed to understand and overcome these challenges as well as optimize pulsing parameters and particle concentrations for optimal ablation efficacy. Potential solutions include developing more stable particles that allow for longer treatments, increasing the concentration of particles, injecting a constant stream of fresh particles, or developing particles that specifically bind to bacteria cells to increase the efficiency of the PMH ablation. However, it is important to note that biofilm removal with PMH is still expected to be clinically useful even if the suspended bacteria are not killed during the treatment since methods for irrigating the catheters after biofilm removal can be utilized to remove the detached bacteria. These methods, which have been previously conducted with urinary catheters [35], would need further investigation for arterial and venous catheter applications to ensure the irrigation of bacteria after PMH does not induce any adverse reactions. Further testing will be needed before the clinical translation of this technology to determine the safety and feasibility of irrigation following biofilm removal with PMH for specific CBMDs.

## 4. Materials and Methods

### 4.1. General Experimental Setup

An 8-element, 1 MHz histotripsy transducer with a geometric focus of 36 mm, an aperture of 5.27 mm, and an f-number of 0.68 was used for all experiments in this study. The full-width half-maximum (FWHM) dimensions at the geometric focus of this transducer were 0.98 mm, 0.93 mm, and 3.9 mm in transverse, elevational, and axial, respectively. The transducer was mounted vertically at the bottom of a tank filled with degassed water. The transducer was driven via a custom high-voltage pulser controlled by a field-programmable gate array (FPGA) board (Altera DE0-Nano Terasic Technology, Dover, DE, USA) programmed to generate single therapy pulses. A computer-guided, 3D positioning system with 0.05 mm motor resolution aligned the hydrophones and/or catheter mimics at the focus of the transducer situated directly between a high-speed camera and a strobe light and automated the precision movements of the catheter mimics during treatments (Figure 1). MATLAB (MATLAB, The MathWorks, Natick, MA, USA) simultaneously controlled the positioning system, camera, and the transducer to ensure accurate positioning, incremental movements, and pulsing. A linear ultrasound imaging probe with a frequency range of 10-18 MHz (L18-10L30H-4, Telemed, Lithuania, EU) was coaxially aligned inside the transducer directly beneath the focus for treatment guidance and monitoring (Figure 1).

### 4.2. Hydrophone Focal Pressure Measurements and Beam Profiles

Focal pressure waveforms for the 1 MHz transducer were measured by a high sensitivity needle hydrophone (HNR-0500, Onda Corporation, Sunnyvale, CA, USA) and a custom-built fiber optic probe hydrophone (FOPH) [36] in degassed water at the focal point of the transducer. Focal pressures were directly measured with the FOPH up to a p− of 21.0 MPa. Higher pressures could not be measured directly due to the risk of damage to the fiber tip from cavitation. Summations of half of the elements within the transducer were used to measure the focal pressures up to 33.4 MPa, following previously used methods [37]. All waveforms were measured using a Tektronix TBS2000 series oscilloscope at a sample rate of 500MS/s, with the waveforms averaged over 128 pulses and recorded in MATLAB.

### 4.3. Cavitation Detection and Treatment Monitoring

High-speed optical imaging and real-time ultrasound imaging were used to capture cavitation activity and monitor histotripsy treatments. Optical imaging was captured by a machine-vision camera (FLIR Blackfly S monochrome, BFS-U3-32S4M-C 3.2 MP, 118 FPS, SonyIMX252, Mono, FLIR Integrated Imaging Solutions, Richmond, BC, Canada) with a global shutter and a 100 mm F2.8 Macro lens (Tokina AT-X Pro, Kenko Tokina Co., LTD, Tokyo, Japan). This combination captured images with a resolution of 3.25 μm per pixel. The catheter samples were backlit by a custom-built, high-speed, pulsed, white-light LED strobe triggered with 4 μs exposures to minimize motion blur of expanding bubbles while providing sufficient lighting through the catheters. All exposures were centered at a delay of 5 μs after the pulse reached the focus allowing for visualization of each cavitation cloud formed. Ultrasound imaging was performed using a portable ultrasound imaging system (SmartUS, Telemed, Lithuania, EU) and a 10-18 MHz linear ultrasound imaging probe (L18-10L30H-4, Telemed, Lithuania, EU). Ultrasound imaging provided real-time cross-sectional monitoring of luminal cavitation. The coaxial ultrasound imaging probe inside the histotripsy therapy transducer and a high-speed optical camera were positioned at 90 degrees from each other, as shown in (Figure 1(a)).

### 4.4. Formulation and Preparation of Gas-Filled Microbubbles

Cationic, lipid-shelled MBs (Figure 1(b)) were synthesized following recently published methods [38]. Briefly, a micellar aqueous mixture of 2 mg/mL 1,2-distearoyl-sn-glycero-3-phosphocholine (DSPC; Avanti Polar Lipids, Alabastar, Alabama), 2 mg/mL polyethylene glycol 6000 monostearate (PEG 6000 MS; Stepan Kessco, Northfield, Illinois), and 0.8 mg/mL 1,2-distearoyl-3-trimethylammonium-propane (DSTAP; Avanti Polar Lipids, Alabastar, Alabama) in 0.9% NaCl (Baxter, Deerfield, Illinois) was prepared by probe-type sonication (20 kHz, 3 min, 50% power, XL2020 instrument, Misonix Inc., Farmingdale, New York). The sonicated medium was filtered through a 0.2 μm Nylon sterile filter, sparged with decafluorobutane gas (DFB, F2 Chemicals Ltd.; Preston, United Kingdom), and then sonicated at the highest power (20 kHz, 30 s) with the same sonicator to generate the MBs. Microbubbles from all samples had mean and median diameters between 1.5 and 2.5 μm with <95.5% of MBs having a diameter less than 5 μm as measured by Coulter Multisizer 3 (Beckman Coulter, Inc., Hialeah, FL) with a 50 μm orifice. MBs were then aliquoted into 13 mm glass vials, which were stoppered for refrigerated storage after filling the headspace with DFB gas. MBs were prepared by removing 1 mL of stock 10^9^ MB/mL into a 2 mL microfuge tube. Serial dilutions were prepared by pipetting 100 μL of 10^9^ MB/mL into 900 μL of degassed 0.9% NaCl in a 2 mL microfuge tube to produce MB concentrations of 10^8^ MB/mL. The same process was repeated to produce the MB 10^7^ MB/mL dilution. MB solutions were stored at 2°C. These concentrations were utilized by starting with stock 10^9^ MB/mL and then reducing the concentration by a factor of 10 until intraluminal cavitation thresholds began to rise.

### 4.5. Formulation and Preparation of PFH Nanocones

PFH-NC’s (Figure 1(c)) were prepared via host-guest interaction between *β*-cyclodextrin (BCD) and perfluorohexane (PFH) with an optimized method similar to the recently published work [28]. Briefly, BCD (100 mg, $8.8\times 10^{-2}$ mmol) was completely dissolved in double-distilled water (6 mL) at room temperature followed by the addition of PFH at the optimized molar ratio of 1 : 5 (BCD : PFH). After overnight stirring, precipitates, which are the NC aggregates that include the inclusion complex of PFH and BCD as building blocks, can be separated by simple filtration or centrifugation and then dried in a vacuum to produce solid white powder. The PFH content of the obtained powder was calculated using gas chromatography through a calibration curve containing different concentrations of free PFH. Further evidence for the presence of the PFH was confirmed using SEM-EDAX analysis. NCs were prepared by weighing out 1.4 mg of PFH NC using (ME104E, Mettler Toledo, Colombus, OH, USA) and diluting into 100 mL of degassed saline 0.9% NaCl in a 300 mL beaker producing a final solution of $6\times 10^{-6}$ mL PFH/mL water. This solution was stirred with a stir bar and placed under vacuum for 30 minutes. After 30 minutes, NCs were serially diluted by pipetting 100 μL of NCs $6\times 10^{-6}$ mL PFH/mL into 900 μL of 0.9% NaCl in a 2 mL microfuge tube to produce NC concentrations of $6\times 10^{-7}$ mL PFH/mL water. The same process was repeated to produce the NC $6\times 10^{-8}$ mL PFH/mL water dilution. NC solutions were stored at 2°C. These concentrations were utilized by starting with a concentration similar to the highest concentration used in previous work done by our lab and then reducing the concentration by a factor of 10 until intraluminal cavitation thresholds began to rise [27].

### 4.6. Cavitation Threshold Experiments

The cavitation threshold was compared for conventional and PMH methods using Tygon catheter mimics cut into 3 cm segments in length and filled with 45 μL of distilled water containing either NCs, MBs, or no particles (0.9% saline). MB concentrations tested were 10^7^, 10^8^, and 10^9^ MB/mL. NC concentrations tested were $6\times 10^{-8}$, $6\times 10^{-7}$, and $6\times 10^{-6}$ mL PFH/mL water. For experiments, the Tygon catheters were fixed to the 3D positioning system above the histotripsy transducer in a tank of degassed water (Figure 1(a)). Real-time ultrasound imaging and high-speed optical imaging were used to capture the resulting cavitation bubble clouds under each set of exposure conditions (n=3). Histotripsy was applied to each sample at a pulse repetition frequency (PRF) of 200 Hz using short single pulses, and the driving voltage of the transducer was slowly increased in 1 V increments (~0.25 MPa) until cavitation was confirmed in the lumen of the catheters on ultrasound imaging and high-speed optical imaging. This process was repeated three times for each sample to determine changes in the cavitation threshold for repeated histotripsy exposures. For each exposure, the lowest p− at which cavitation was observed in each catheter was recorded as the cavitation threshold. The process was repeated for three exposures, with the cavitation thresholds recorded as exposure 1, exposure 2, and exposure 3, respectively. Statistical analysis was conducted by determining the average and 95% confidence interval for each of the three exposures (n=3 catheters per experimental condition). Further, two-way ANOVA testing with post hoc Tukey’s was used to determine inter and intra group statistical significance. Finally, another set of threshold experiments were conducted to test the changes in cavitation threshold for catheters exposed to full histotripsy treatment scans (Supplemental).

### 4.7. Selective Luminal Cavitation Using Particle-Mediated Histotripsy

Experiments were conducted to demonstrate the feasibility of selectively generating histotripsy cavitation inside catheter lumens using a PMH approach. Tygon catheters were prepared, the same as the previous threshold experiments with 45 μL of 10^9^ MB/mL, $6\times 10^{-6}$ mL PFH/mL water of NCs, or 0.9% saline. Coaxial ultrasound imaging transducer and optical imaging were used to position the focus of the treatment transducer in the center of the catheter lumen approximately halfway in length. Histotripsy pulses were applied to the catheters at a p− of 12.3 MPa or 29.8 MPa at 200 PRF, corresponding to pressures both above and below the conventional histotripsy cavitation threshold. Histotripsy pulses were applied to the catheter lumen for 15 seconds, and the particle-filled catheters were then moved vertically in 3 mm increments every 5 seconds followed by the acquisition of additional optical and ultrasound images at each location to compare cavitation generation in regions inside the catheter lumen to those outside of the catheter in the surrounding media.

### 4.8. Culturing a Luminal Biofilm inside Tygon Catheter Mimic

A biofilm was cultured on the inner lumen of the as previously conducted in our previous study [15]. Briefly, Tygon catheter mimics with an inner diameter of 1/16${}^{\prime \prime }$ ID and a wall thickness of 1/16${}^{\prime \prime }$ (McMaster Carr, Part # 5894K31) were selected to provide a model system with similar dimensions and material properties to clinically used catheters. The Tygon tubing was cut into 3 cm long catheter segments and soaked in 10% bleach for 15 minutes for sterilization. Silicone stoppers were also sterilized via this same process. After sterilization, segments and stoppers were washed with sterile distilled water (diH2O) twice. Once washed, sterilized catheters and stoppers were placed on a sterile petri dish to dry prior to inoculation with *P. aeruginosa* (PA14). PA14 was subcultured from frozen stock stored at -80°C. Luria-Bertani (LB) agar plates were streaked with a sterile inoculating loop dipped in a frozen PA14 aliquot and incubated overnight at 37°C for 12 hours. After overnight culture, a single colony was suspended in a 10 mL culture tube filled containing 4 mL of sterile LB broth. The culture tube was incubated and shook at 37°C at 175 RPM for 12 hours. Bacterial density was standardized using Spectrophotometry (Biomate 5 Spectrophotometer, Thermo Fisher, Waltham, MA) at Optical Density 600 nm (OD600) to A=0.01. Overnight cultures were diluted into fresh LB to produce 5 mL of PA14 standardized to A=0.01, and the initial bacterial concentration was confirmed by 10-fold serial dilution. An aliquot of 1 mL of LB and PA14 standardized to A=0.01 was used to pipet 45 μL of standardized PA14 into the lumen of the sterilized Tygon catheters which were then caped on one end. The same process was repeated for catheters of sterile LB as a control. Capped catheters were placed in a 96 well plate (stopper side down) and incubated in a humidified incubator at 25°C for 48 hours. After 48 hours to grow biofilm in static cultures, Tygon catheters were flushed 2 times with 1 mL of sterile LB followed by 2 times with 1 mL of diH2O to remove planktonic bacteria. Catheters were then subject to subsequent histotripsy treatment.

### 4.9. Particle-Mediated Histotripsy Biofilm Removal

Tygon catheter mimics with cultured biofilms were used to investigate the feasibility of PMH for removing luminal biofilms. Tygon catheters were split into a NC group and a MB group each with separate control groups. Samples for each consisted of untreated (n=6), treated (n=6), background (n=6), and LB control (n=3) groups. The NC group treatment and control were filled with 45 μL of 0.9% saline solution containing $6\times 10^{-6}$ mL PFH/mL, while the MB group treatment and control filled with 45 μL of 10^9^ MB/mL. The samples were then capped on each end by silicone stoppers. Histotripsy treatment of the Tygon catheters was conducted by fixing the catheters to a 3D positioning system via a 3D printed scaffold (Figures 1(a) and 1(d)). A predetermined treatment zone of 15.5 mm was selected to cover the area between the silicone stoppers (Figures 1(e) and 1(f)). The focus of the transducer was aligned 5 mm inside of the first silicone stopper, and a custom program was used to move the focus across the total treatment zone in 1 mm steps. Histotripsy was applied for 2.5 seconds in each location at a p− of 12.3 MPa for the NC group and 12.3 MPa for the MB group at a PRF of 200 Hz (500 pulses per location), resulting in a total treatment time of 6 minutes and 27.5 seconds per scan of the catheter. Cavitation was monitored during the treatment using both ultrasound and optical imaging. Untreated control catheters underwent this same process with the treatment transducer turned off. After treatment, catheters were flushed twice with sterile LB and twice more with diH2O to remove planktonic bacteria. Catheters were then air-dried for 10 minutes, and 45 μL of 0.1% crystal violet (CV) stain was pipetted into the lumen of the catheter containing the biofilm to be quantified and left for 10 minutes. Catheters were then flushed 3 times with 1 mL of diH2O. Photographs of CV-stained biofilms were then taken. CV within the catheter was then solubilized with 100 μL of 33% glacial acetic acid into 96 well flat-bottom plates for spectrophotometry analysis (Spectra Max Plus 384 Microtiter Plate Reader, Molecular Devices, San Joes, CA). Each well was read at an absorbance of OD590 nm zeroed to 100 μL of 33% glacial acetic acid, and the resulting optical density data was recorded. Absorbance of background wells of 100 μL sterile LB was also included. Statistical analysis was conducted using Student’s t-tests, with corrected p values < 0.05 considered significant.

### 4.10. Particle-Mediated Histotripsy Bactericidal Effects

In addition to the removal of adherent luminal biofilms, a final set of experiments was conducted to investigate the dose-dependent bactericidal effect of PMH on PA14 bacteria in suspension within Tygon catheters. Although our recent study demonstrated histotripsy could kill luminal bacteria, previous work has also shown that particle-mediate histotripsy methods are less efficient at ablating tissue, demonstrating the need for additional investigation into the feasibility of using PMH for killing bacteria [33]. To test this, Tygon catheters containing suspended PA14 standardized to A=0.01, as previously described in the biofilm culture section, were treated directlyrather than suspended bacteria being used for biofilm formation. Tygon catheters filled with suspended PA14 were split into PMH treated (n=3) and untreated (n=3) groups using the same concentrations and experimental set-up described in the previous section. The NC treatment and control were filled with 22.5 μL of 0.9% saline solution containing $1.2\times 10^{-5}$ mL PFH/mL water and 22.5 μL of standardized PA14 for a final concentration of $6\times 10^{-6}$ mL PFH/mL water, while the MB group treatment and control were filled with 22.5 μL of 0.9% saline solution containing $2\times 10^{9}$ MB/mL and 22.5 μL of standardized PA14 for a final concentration of 10^9^ MB/mL and capped on each end by silicone stoppers. Histotripsy was applied at a PRF of 200 Hz for 2.5 seconds per treatment location (500 pulses) at a p− of 12.3 MPa for both groups, resulting in a treatment time of 6 minutes and 27.5 seconds per scan of the catheter. Cavitation was confirmed throughout the treatment process using ultrasound and optical imaging, with control catheters again undergoing the same process with the treatment transducer turned off but filled with respective NCs or MBs. Treated catheters were further split into groups receiving 1, 2, 4, and 6 treatment scans to quantify the dose-dependent bactericidal effect of PMH. Each consecutive treatment scan was conducted by repositioning the treatment focus at the initial start point and restarting the automated treatment, as described above.

After treatment, catheters were uncapped, and the 45 μL within the lumen was resuspended in 1 mL of diH20 and termed stock concentration. Stock concentrations were serially diluted by removing 100 μL and pipetting it into 900 μL of diH2O. This was also conducted for the separate initial group that was set aside after the standardization of PA14 to A=0.01. Serial dilutions were carried out from stock concentration to 10^-7^ for a total of 8 serial dilutions. 100 mm LB agar plates were divided into 8 sections. Within each section, 20 μL of the various serial dilutions were plated using a previously described protocol [39, 40]. PA14 plated agar plates were then incubated at 37°C for 24 hours. After incubation, colonies were counted for each serial dilution that had a countable number of colonies. If two serial dilutions were countable, they were averaged together. The number of CFU/mL for the stock concentration was determined by n×5×10d+1 where d=0. The number of CFU/mL for each serial dilution was calculated by manipulating the variable d. An additional 22.2 was factored into the control and treatment groups which corrected for the initial dilution of 45 μL in the Tygon catheters into 1 mL of diH2O. Further, an additional multiplication of 2 was added to correct for the dilution of the suspended bacteria with each of NC and MB. CFU/mL within the 45 μL Tygon catheters were then finally determined by using the equation $2\times \left(n\times 22.2\times 5\times 10d+1\right)$. CFU counts were recorded for each catheter and CFU/mL calculated using the above formulas. Statistical analysis was conducted using Student’s t-tests, with experiment-wide Bonferroni correction, between respective treatment and control groups with corrected p values < 0.05 considered significant.

## Supplementary Materials

Supplementary Materials Supplemental 1: optical and ultrasound images after increasing scan number. Optical and ultrasound images showing reduction in cavitation cloud size and intensity as scan number increases. Particle concentrations used, MB (109 Bubble/mL) and NC ($6\times 10^{-6}$ PFH mL/mL water) concentrations. Supplemental 2: MB and NC threshold after increasing scan number. Cavitation threshold tested after various scans through a catheter mimic with 155 points in 0.1 mm increments at 500 pulses per point at 12.3 MPa pressure during each respective scan.
