# Supplementary material for: Particle-Mediated Histotripsy for the Targeted Treatment of Intraluminal Biofilms in Catheter-Based Medical Devices
Source: BME Front. 2022 Jul 5;2022:9826279. doi: 10.34133/2022/9826279 (PMC10521694; doi:10.34133/2022/9826279)
Supplement: Supplementary Materials — Supplemental 1: optical and ultrasound images after increasing scan number. Optical and ultrasound images showing reduction in cavitation cloud size and intensity as scan number increases. Particle concentrations used, MB (109 Bubble/mL) and NC (6×10^−6^ PFH mL/mL water) concentrations. Supplemental 2: MB and NC threshold after increasing scan number. Cavitation threshold tested after various scans through a catheter mimic with 155 points in 0.1 mm increments at 500 pulses per point at 12.3 MPa pressure during each respective scan. [file 9826279.f1.zip › Supplemental 2.pptx]

## Slide 1
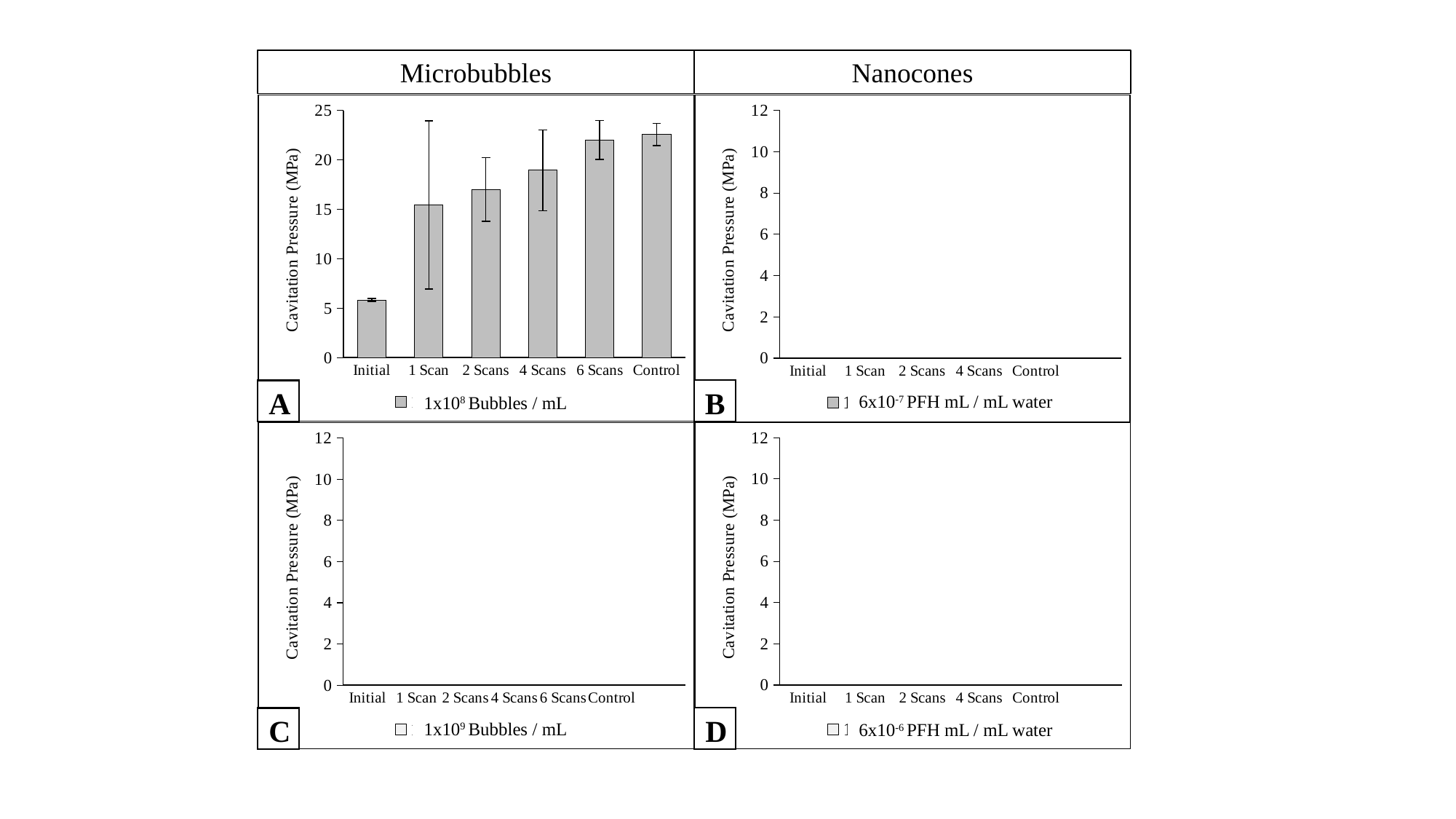

Microbubbles
Nanocones
### Chart
| Category | 1x10^8^ |
|---|---|
| Initial | 5.82562584 |
| 1 Scan | 15.42071821333333 |
| 2 Scans | 17.001727026666664 |
| 4 Scans | 18.93944377 |
| 6 Scans | 21.996909466666665 |
| Control | 22.563664756666668 |
### Chart
| Category | 1x10^-5 |
|---|---|
| Initial | 11.262826650000001 |
| 1 Scan | 17.225928156666665 |
| 2 Scans | 20.33526045 |
| 4 Scans | 22.10473344 |
| Control | 22.563664756666668 |B
A
6x10^-7^ PFH mL / mL water
1x10^8^ Bubbles / mL
### Chart
| Category | 1x10^-4 |
|---|---|
| Initial | 10.19024183 |
| 1 Scan | 10.693908200000001 |
| 2 Scans | 20.140959136666666 |
| 4 Scans | 21.569345439999996 |
| Control | 22.563664756666668 |
### Chart
| Category | 1x10^9^ |
|---|---|
| Initial | 5.22964 |
| 1 Scan | 10.401425816666665 |
| 2 Scans | 13.04208527 |
| 4 Scans | 14.88149955 |
| 6 Scans | 17.72849595 |
| Control | 22.563664756666668 |D
C
1x10^9^ Bubbles / mL
6x10^-6^ PFH mL / mL water
