# Supplementary material for: Particle-Mediated Histotripsy for the Targeted Treatment of Intraluminal Biofilms in Catheter-Based Medical Devices
Source: BME Front. 2022 Jul 5;2022:9826279. doi: 10.34133/2022/9826279 (PMC10521694; doi:10.34133/2022/9826279)
Supplement: Supplementary Materials — Supplemental 1: optical and ultrasound images after increasing scan number. Optical and ultrasound images showing reduction in cavitation cloud size and intensity as scan number increases. Particle concentrations used, MB (109 Bubble/mL) and NC (6×10^−6^ PFH mL/mL water) concentrations. Supplemental 2: MB and NC threshold after increasing scan number. Cavitation threshold tested after various scans through a catheter mimic with 155 points in 0.1 mm increments at 500 pulses per point at 12.3 MPa pressure during each respective scan. [file 9826279.f1.zip › SUPPLEMENTARY MATERIALS.docx]

SUPPLEMENTARY MATERIALS

**BRIEF SESCRIPTION**

Due to the results of the bactericidal experiments which showed only a small amount of cell death after six scans and an observable reduction in cavitation activity over the course of the treatments, a set of experiments was conducted to investigate the change in the histotripsy cavitation threshold after 6 independent scans through the catheter lumen. Optical images of MMH showed cavitation consistent through scans one and two however during scans four and six minimal posterior wall cavitation was seen **(Fig. S1)**. Similarly, optical images of NMH showed cavitation consistent through scans one and two however no cavitation is seen in subsequent scans.

**RESULTS**

*Particle-Mediated Thresholds After Increasing Scan Number*

Prior to any treatment, initial results showed MB-filled catheters had a cavitation threshold of 5.2±0.3 and 5.8±0.1 MPa for the MB concentrations 1×10^8^ and 1×10^9^ MBs per mL, respectively **(Fig. S2 A&C)**. After the first scan, the cavitation threshold increased to 10.4±5.4, and 17.0±8.5 MPa for 1×10^8^ and 1×10^9^ MBs per mL concentrations, respectively **(Fig. S2 A&C)**. After two scans, the cavitation threshold increased further to 13.0±3.5, and 17.0±3.2 MPa for the MB concentrations 1×10^8^ and 1×10^9^ MBs per mL, respectively **(Fig. S2 A&C)**. After 4 scans the cavitation threshold continued to increase to 14.9±5.6, and 18.9±4.1 MPa for the MB concentrations 1×10^8^ and 1×10^9^ MBs per mL, respectively **(Fig. S2 A&C)**. Finally, after 6 scans the cavitation threshold approached that of the control catheters at 17.7±7.4, and 22.0±2.0 MPa for the MB concentrations 1×10^8^ and 1×10^9^ MBs per mL, respectively **(Fig. S2 A&C)**. For comparison, control catheters with no MBs showed a cavitation threshold of 22.6±1.1MPa **(Fig. S2 A&C)**, demonstrating that the cavitation threshold returned nearly equivalent levels to the controls for MB samples after six scans. This result suggests that MBs were likely destroyed during the treatment and were no longer capable of reducing the histotripsy threshold.

Prior to any treatment, initial results showed NC-filled catheters had a cavitation threshold of 10.2±0.2, and 11.3±0.3 MPa for the NC concentrations 6x10^-6^ and 6x10^-7^ mL PFH per mL water, respectively **(Fig. S2 B&D)**. After the first scan, the cavitation threshold increased to 10.7±0.1, and 17.2±3.3 MPa for the NC concentrations 6x10^-6^ and 6x10^-7^ mL PFH per mL water respectively **(Fig. S2 B&D)**. After two scans, the cavitation threshold increased further to 20.1±3.9, and 20.3±1.6 MPa for the NC concentrations 6x10^-6^ and 6x10^-7^ mL PFH per mL water, respectively **(Fig. S2 B&D)**. Finally, after 4 scans the cavitation threshold continued to increase to 21.6±1.1, and 22.1±0.2 MPa for the NC concentrations 6x10^-6^ and 6x10^-7^ mL PFH per mL water, respectively **(Fig. S2 B&D)**. Control catheters showed a cavitation threshold of 22.6±1.1MPa thus NCs were not tested beyond four scans **(Fig. S2 B&D)**. As seen with the MBs cavitation became inconsistent beyond one scan for the NC concentration 6x10^-7^ mL PFH per mL water and two scans for 6x10^-6^ mL PFH per mL water.

**DISCUSSION**

Results from these experiments demonstrate an increase in the cavitation threshold with each increase in the number of scans through the catheter, likely due to the destruction of the particles after being subjected to the histotripsy bubble clouds. These data did not match the results from our initial cavitation threshold experiments that only showed an increased threshold for the lowest particle concentrations and only showed an increase in the cavitation threshold after subsequent exposures for the MB samples. However, the results from these longer treatments in which the entire catheter was exposed to multiple histotripsy treatment scans showed a greater increase in the cavitation threshold. This finding is likely due to the destruction of more MBs and NCs over the longer treatment duration, as would be expected. Overall, these results help to explain the lack of bactericidal effect observed after multiple treatment scans and demonstrate the need for further work improving PMH methods to provide sustained cavitation and bacteria ablation.

**METHODS**

A set of threshold experiments was conducted to test the changes in cavitation threshold for catheters exposed to multiple histotripsy treatment scans across the catheter. MB concentrations tested included 10^9^ and 10^8^ MB/mL 0.9% saline while NC concentrations tested included 6x10^-6^ and 6x10^-7^ mL PFH /mL water. Control catheters were filled with no particles (0.9% saline). These experiments were conducted by treating particle-filled catheters with a custom treatment program that applied 500 histotripsy pulses per point over a predetermined 155 points in 0.1 mm increments from left to right at a pressure of 12.3 MPa at 200 PRF. After each scan, the focus was moved 7.75 mm to the left resulting in a centered focus with respect to the catheters length. The cavitation threshold was again tested using ultrasound and high-speed optical imaging, as described in the previous Methods section.
